# Supplementary material for: An atlas of gene regulatory elements in adult mouse cerebrum
Source: Nature. 2021 Oct 6;598(7879):129–36. doi: 10.1038/s41586-021-03604-1 (PMC8494637; doi:10.1038/s41586-021-03604-1)
Supplement: Supplementary file 1 — This file contains Supplementary Notes. [file 41586_2021_3604_MOESM1_ESM.pdf]

nature portfolio

https://doi.org/10.1038/s41586-021-03604-1

Supplementary information

An atlas of gene regulatory elements in adult mouse cerebrum

In the format provided by the authors and unedited

SUPPLEMENTARY NOTES

Rigorous analysis of single nuclei chromatin accessibility of the adult mouse brain

The snATAC-seq libraries from 45 dissected region were sequenced, and the reads were deconvoluted based on nucleus-specific barcode combinations and sequencing reads showed nucleosome-like periodicity (Extended Data Figure 2a-e). Excellent correlation between datasets from similar brain regions (0.92-0.99 for isocortex; 0.89-0.98 for OLF; 0.79-0.98 for CNU; 0.88-0.98 for hippocampus) and between biological replicates (0.98 in median, range from 0.95 to 0.99) indicated high reproducibility and robustness of the experiments (Extended Data Figure 2f). We confirmed that the dataset of each replicate met the quality control metrics (Extended Data Figure 2g-k, see **Methods**). We selected nuclei with at least 1,000 sequenced fragments that displayed high enrichment (>10) in the annotated transcriptional start sites (TSS; Extended Data Figure 2g). We also removed the snATAC-seq profiles likely resulting from potential barcode collision or doublets using a procedure modified from Scrublet[66] (Extended Data Figure. 2h, see **Methods**). Altogether, 28.7% nuclei were deemed low-quality, and an additional 6.2% nuclei potential doublets (Extended Data Figure 2i). In total of 813,799 nuclei passed rigorous quality control filtering.

Robust clustering based on accessible chromatin

To determine number of cell types within each subclass, we evaluated the relative stability from a consensus matrix based on 300 rounds of clustering with randomized starting seed at each resolution. Then, we calculated the proportion of ambiguous clustering (PAC) score and dispersion coefficient (DC) to find the optimal resolution (local minimum and maximum) for cell type clustering (Extended Data Figure 3b-g, see **Methods**).

The clustering result of snATAC-seq was robust to variation of sequencing depth, signal-to-noise ratios, and showed no batch effect from biological replicates demonstrated using both the K-nearest neighbor batch effect test (kBET) and local inverse Simpson's index (LISI) analysis (Extended Data Figure 4). In addition, the cellular composition of different

brain dissections was highly reproducible between the two biological replicates (Extended Data Figure 5).

Cell type proportions are comparable across experimental platforms

To test if the cell type proportion measurements estimated based on clustering of snATAC-seq data were robust and reliable, we performed snATAC-seq using a droplet-based platform from 10x Genomics (10x) for two biological replicates of the primary motor cortex (dissected region: 3C). The numbers of nuclei passing quality control for both methods were comparable (combinatorial barcoding: 15,939 nuclei, 10x: 16,314, Extended Data Figure 8a). Co-embedding of all datasets showed that the chromatin accessibility profiles and cell clusters from both platforms were in excellent agreement across cell types (Extended Data Figure 8a-c). This was further shown by a confusion matrix comparing the similarity between clusters derived from the combinatorial barcoding and the 10x platform, respectively (Extended Data Figure 8d). Further, we did not observe a significant difference in cell type composition between the two platforms (Extended Data Figure 8e), except for one small population of vascular cells (VLMC, 326 nuclei from 10x, 155 from sci).

Comparison of cell clusters between snATAC and scRNA-seq

To directly compare our single nucleus chromatin accessibility derived cell clusters with the single cell transcriptomics defined taxonomy of the mouse brain[2], we performed integrative analysis with single cell RNA-seq using Seurat 3.0[75] (RRID:SCR_016341). For 155 of 160 cell types defined by snATAC-seq (A-Type), we could identify a corresponding cell cluster defined using scRNA-seq data (T-Type; overlap score cut-off at 0.5; Extended Data Figure 10a, b); conversely, for 84 out of 100 T-types we identified one, or in some cases more, corresponding A-types (Extended Data Figure 10a, c). Of note, two clusters fell into different classes. The Cajal-Retzius cells (CRC) were part of the GABAergic class in A-type but glutamatergic class in T-type and one small non-neuronal A-type cluster,

VPIA3 (Vascular and leptomeningeal like cells) co-clustered with CRC T-type (Extended Data Figure 10a, Supplementary Table 5).

Regional specificity in different brain cell types

The single cell atlas of chromatin accessibility generated in this study provides a unique opportunity to characterize the cellular composition of each brain area and the gene regulatory programs within each constituent cell type that underlay its specialized functions.

Most glia cell types were ubiquitously distributed throughout the different brain dissections and showed very low regional specificity (Fig. 1f), with the exception of neuronal intermediate progenitor cells (NIPC) and radial glia-like cells (RGDG, RGSZ), which are restricted to dissections of lateral ventricles and dentate gyrus (DG) that contain the two main neurogenic niches in the mouse brain, the subventricular zone (SVZ) and the subgranular zone (SGZ) of the dentate gyrus (DG) in the hippocampus[42] (Extended Data Figure 12). Additionally, an astrocyte cell type (ASCN) is localized exclusively to pallidum and lateral septal complex (Extended Data Figure 12). In contrast to the glia cell types, most GABAergic and glutamatergic neurons showed a significant regional specificity (Fig. 1f, g). Glutamatergic neurons showed slightly higher regional specificity than GABAergic neurons, consistent with previous single cell transcriptomic analysis (Fig. 1g, lower panel)[4]. We found a stark separation based on brain sub-regions for distinct cell types of glutamatergic neurons, including the granular cell (DGGR) which was restricted to the dentate gyrus (DG) of the hippocampus, the CA1GL and CA3GL in the cornu ammonis field (CA) of the hippocampus, and PIRGL, OLFGL, and OBGL in the olfactory area (Fig. 1c, f). While some cell types of GABAergic neurons were broadly distributed, many others showed strong regional specificity. For example, the matrix D1 neurons (MXD, Extended Data Figure 14a, Supplementary Table 5) were found exclusively in the pallidum (PAL, Extended Data Figure 14a, b), an observation that was corroborated by *in situ* hybridization (ISH) data from the Allen Brain Atlas (ABA)[44] for the gene *Isl1* that displayed high accessibility exclusively in MXD neurons (Fig. 1c, f, Extended Data Figure 14c,d).

The GABAergic and glutamatergic neuron cell types were highly restricted to individual brain regions or dissections (Fig. 1g, lower panel). For example, the PVGA7 cell type was restricted to the nucleus accumbens (ACB) and caudoputamen (CP) (Extended Data Figure 14e, f). This type of GABAergic neurons showed high accessibility for *Kit* and *Pde3a*, genes that are highly expressed in striatal parvalbumin interneurons[109] (Extended Data Figure 14g) and in the caudoputamen and nucleus accumbens as evidenced by *in situ* hybridization (ISH)[44] (Extended Data Figure 14h). Our analysis also revealed cortical layer-specific intra-telencephalic (IT) neurons which were restricted to distinct regions - one type of IT neurons from cortical layer 4 (ITL4GL1) was located in the primary and secondary somatosensory area (SSp, SSs), another type of IT neuron from cortical layer 5 (ITL5GL3) was restricted in the anterior cingulate area (ACA). Notably, these results were consistent with independent findings from DNA methylation profiles from the same dissections (companion paper, Liu, Zhou et al.[29]). Furthermore, some cell types showed differences across dissections along the anterior-to-posterior axis within one functional brain region. For example, in the cornu ammonis field 1 (CA1) of the hippocampus, one sub-type (CA1GL) with high accessibility at the *Dcn* gene locus was restricted to posterior dissections (ventral, CA-3 and CA-4; Extended Data Figure 14i-k). This trend derived from chromatin accessibility profiles was further supported by detection of *Dcn* expression in posterior parts of CA1 (Extended Data Figure 14l, m) consistent with previous reports on an expression gradient of *Dcn* in CA1[110].

Identification of reliable and reproducible cCREs in different mouse brain cell types

We aggregated the snATAC-seq profiles from the nuclei comprising each cell cluster/type and determined the open chromatin regions with MACS2[30] (Extended Data Figure 15a). We then selected the genomic regions mapped as accessible chromatin in both biological replicates, finding an average of 93,775 (range from 50,977 to 136,962) sites (500-bp in length) in each cell type (Extended Data Figure 15b). We found that read depth or cluster size can affect MACS2 peak calling scores due to the nature of the Poisson distribution test in MACS2, which will introduce bias when we apply a constant cutoff. Ideally, we

would perform a reads-in-peaks normalization between clusters, but in practice, this type of normalization is not possible because we do not know how many peaks are accessible in each cell cluster. For these reasons, we used “score per million” (SPM) to correct for this issue (Extended Data Figure 15c, see **Methods**).

Transcription factor motifs enriched in cell type restricted cCRE modules

We observed that the majority of cCREs displayed highly variable levels of chromatin accessibility across cell types and could be grouped into modules. These cell-type restricted modules were enriched for transcription factor motifs recognized by known transcriptional regulators such as the SOX family factors in module M40 for oligodendrocytes (OGC, Supplementary Table 11)[61,111]. We also found strong enrichment for the known olfactory neuron regulator LIM homeobox factor LHX2 in module M5 which was associated with GABAergic neurons in the olfactory bulb (OBGA1; Supplementary Table 11)[112].

Open chromatin regions characterize distinct medial septal nucleus neuron cell types

In the medial septal nucleus neurons (MSGA), we identified a total of 46,453 cCREs that showed cell type restricted chromatin accessibility (Extended Data Figure 17d-g). One cell type (MSGA10) corresponded to cholinergic neurons (Supplementary Table 5, Extended Data Figure 17h, i) and the cCREs showing cell-type-specific accessibility in this cell type were enriched for ISL1 and NKX2-1, two known transcriptional regulators of cholinergic neurons[113,114] (Extended Data Figure 17j, Supplementary Table 12). Interestingly, prenatal deletion of *Nkx2-1* leads to a nearly complete loss of cholinergic neurons in the basal ganglia[113]. *Isl1* was shown to have a critical role in the lineage determination of cholinergic neurons during forebrain development[114].

Open chromatin regions characterize astrocyte cell types from distinct brain regions

For the astrocyte cell type ASCG, we identified regional-specific cCREs (Extended Data Figure 16c, d) with enrichment of distinct transcription factor motifs (Extended Data Figure 18k, l; Supplementary Table 15). For example, motifs for HMG-factors Tcf7 and LEF1 were enriched in open chromatin of ASCG from caudoputamen (CP), ROR nuclear receptors in open chromatin of ASCGs in the somatosensory cortex (SSp and SSs) and homeobox transcription factors in open chromatin of ASCGs from dentate gyrus (DG) and caudoputamen (CP, Extended Data Figure 18l; Supplementary Table 15).

Characterization of enhancer-gene pairs

The median distance between the putative enhancers and the target promoters was 178,911 bp (Extended Data Figure 19a). Each promoter region was assigned to a median of 7 putative enhancers (Extended Data Figure 19b), and each putative enhancer was assigned to one gene on average.

Enhancer-gene pairs active in limited number of cell types in the mouse brain

The majority of modules of enhancer-gene pairs were active in a limited number of cell-types or even cell-type specific. For example, module M33 was associated with perivascular microglia (PVM). Genes linked to putative enhancers in this module were related to immune processes and the putative enhancers were enriched for the binding motif for ETS-factor PU.1, a known master transcriptional regulator of this cell lineage (Fig. 4c, d, Supplementary Table 17, 19 and 20)[115]. Similarly, module M35 was strongly associated with oligodendrocytes (OGC) and the putative enhancers in this module were enriched for motifs recognized by the SOX family of transcription factors (Fig. 4c, d, Supplementary Table 17 and 20)[111]. We also identified module M15 associated with several cortical glutamatergic neurons (IT.L2/3, IT.L4, IT.L5/6, IT.L6), in which the putative enhancers were enriched for sequence motifs recognized by the bHLH factors

NEUROD1 (Fig. 4c, d, Supplementary Table 17 and 20)[116]. Another example was module M10 associated with medium spiny neurons (MSN1 and 2), in which putative enhancers were enriched for motifs for the MEIS factors, which play an important role in establishing striatal inhibitory neurons (Fig. 4c, d, Supplementary Table 17 and 20)[117]. Notably and in stark contrast to the cell-type specific patterns at putative enhancers, the chromatin accessibility at promoter regions showed little variation across cell types (Extended Data Figure 19c). This is consistent with the paradigm that cell-type-specific gene expression patterns are largely established by distal enhancer elements[104,118].

Distinct groups of transcription factors are implicated at the enhancers and promoters in the pan-neuronal module

We identified one module of gene-enhancer pairs (M1) that was active across neuronal clusters and strongly enriched for CTCF, RFX and MEF binding sites (Supplementary Table 21). The role of CTCF at M1 cCREs in neuronal cells was supported by two lines of experimental evidence. First, 80.4 % of cCREs with a predicted CTCF binding motif in M1 were bound by CTCF, evidenced by reanalysis of previously published CTCF ChIP-seq data of the adult mouse cortex[31] (Extended Data Figure 20a). Second, we found that 13.5% of these CTCF-bound cCREs were in spatial proximity with the predicted target gene promoters in neuronal cells in the mouse hippocampus, as evidenced by chromatin loops detected from single nucleus chromatin organization analysis (snm3C-seq[119] and companion paper Liu, Zhou et al.[29], Extended Data Figure 20b, c), while just 7.8% were expected by random chance (p-value = 0.0044, Fisher's exact test). For example, we found one CTCF peak overlapping a distal cCRE positively correlated with expression of *Nsg2*, which is one of the most abundant proteins in the nervous system during perinatal development, and is required for normal synapse formation and/or maintenance[120] (Extended Data Figure 20d).

The RFX family of transcription factors are best known to regulate the genes involved in cilium assembly pathways[121]. The RFX binding motif was strongly enriched at the putative enhancers for genes encoding proteins that participate in postsynaptic transmission,

postsynaptic transmembrane potential, mitochondrion distribution, and receptor localization to synapse (Extended Data Figure 19f, Supplementary Table 22). For example, we found the RFX motif in a distal cCRE positively correlated with expression of *Kif5a* which encodes a protein essential for $GABA_A$ receptor transport (Extended Data Figure 19g)[122]. This observation thus suggests a role for RFX family of transcription factors in regulation of synaptic transmission pathways in mammals. Similar to CTCF and RFX, the MEF2 family transcription factors have also been shown to play roles in neurodevelopment and mental disorders[123]. Consistent with this, the genes associated with putative enhancers containing MEF2 binding motifs were selectively enriched for those participating in positive regulation of synaptic transmission, long-term synaptic potentiation, and axonogenesis (Extended Data Figure 19f, Supplementary Table 22). For example, we found a distal cCRE harboring a MEF2 motif positively correlated with expression of *Cacng2* which encodes a calcium channel subunit that is involved in regulating gating and trafficking of glutamate receptors (Extended Data Figure 19h)[124]. Notably, in cell types with high chromatin accessibility, cCREs and promoters of putative target genes also showed low levels of DNA methylation (Extended Data Figure 19g, h, see companion manuscript by Liu, Zhou et al.[29]).

Interestingly, motif analysis of promoters of genes linked to cCREs in the module M1 revealed the potential role of very different classes of transcription factors in neuronal gene expression. Among the top-ranked transcription factor motifs are those recognized by CREB (cAMP-response elements binding protein), NF-κB, STAT3 and CLOCK transcription factors (Supplementary Table 23). Enrichment of CREB binding motif in module M1 gene promoters is consistent with its well-documented role in synaptic activity-dependent gene regulation and neural plasticity[125,126]. Enrichment of NF-κB[127], STAT3[128] and CLOCK[129] binding motifs in the module M1 gene promoters is interesting, too, as it suggests potential roles for additional extrinsic signaling pathways, i.e. stress, interferon, circadian rhythm, respectively, in the regulation of gene expression in neurons.

Interpreting noncoding variants associated with neurological traits and diseases

Most variants are located in noncoding parts of the genome that often lack functional annotations[50]. Even when a noncoding regulatory sequence is annotated as cCREs, the cell-type specificity is often not known because previous bulk assays employed heterogeneous tissues and yielded only population average signals[130,131]. We performed linkage disequilibrium score regression (LDSC)[52] analysis to determine if genetic heritability of non-neuropsychiatric traits is enriched for SNPs within cCREs (see **Methods,** Supplementary Table 25). CCREs of non-neuronal mesenchymal cells were not enriched for neurological traits but showed enrichment for cardiovascular traits such as coronary artery disease (Fig. 5). Similarly, variants associated with height were also significant in these cell types (Fig. 5). CCREs in microglia were significantly enriched for variants related to immunological traits like inflammatory bowel disease, Crohn's disease and multiple sclerosis (Fig. 5).

To further demonstrate how cell-type resolved maps of cCREs help interpret noncoding disease risk variants, we focused on those variants associated with schizophrenia (SCZ). We obtained 4,356 likely SCZ causal variants with a posterior probabilities of association (PPA) score greater than 1% based on Bayesian fine-mapping[132] determined by the Psychiatric Genomics Consortium (https://www.med.unc.edu/pgc/). For 37.6% (1,639/4,356) we identified the homologous sequences in the mouse genome. 26.9% (441/1,639) of these reside in mouse cCREs defined in the current study, significantly higher than the expected rate of 5.9% when a similar number of elements were randomly selected from all the previously annotated mouse cCREs[104] (*p*-value < 0.00001, Fisher's exact test). 206 of the cCREs containing one or more potential causal SCZ risk variants could be linked 98 putative target genes. For example, in one schizophrenia-associated locus[97] (Extended Data Figure 22b), four cCREs containing five potential causal variants (rs982085, rs13164092, 5:137841064, 5:137932167, 5:137947196) displayed accessibility in multiple neuronal cell types, including Cajal Retzus cells (CRC), a cell type that has been implicated in schizophrenia[133] (Extended Data Figure 22b). One of these cCREs (containing rs13164092, 5:137841064) overlapped a forebrain enhancer[108] that

was predicted to regulate the expression of multiple SCZ associated genes, including *Fam53c*, *Reep2*[134]. These observations provide new hypotheses regarding the potential functions of noncoding SCZ risk variants.

SUPPLEMENTARY REFERENCES

109. Enterria-Morales, D. et al. Molecular targets for endogenous glial cell line-derived neurotrophic factor modulation in striatal parvalbumin interneurons. *Brain Commun.* **2**, fcaa105 (2020).

110. Harris, K. D. et al. Classes and continua of hippocampal CA1 inhibitory neurons revealed by single-cell transcriptomics. *PLoS Biol.* **16**, e2006387 (2018).

111. Glasgow, S. M. et al. Mutual antagonism between Sox10 and NFIA regulates diversification of glial lineages and glioma subtypes. *Nat. Neurosci.* **17**, 1322–1329 (2014).

112. Kolterud, A., Alenius, M., Carlsson, L. & Bohm, S. The Lim homeobox gene Lhx2 is required for olfactory sensory neuron identity. *Development* **131**, 5319–5326 (2004).

113. Magno, L. et al. The integrity of cholinergic basal forebrain neurons depends on expression of Nkx2-1. *Eur. J. Neurosci.* **34**, 1767–1782 (2011).

114. Cho, H. H. et al. Isl1 directly controls a cholinergic neuronal identity in the developing forebrain and spinal cord by forming cell type-specific complexes. *PLoS Genet.* **10**, e1004280 (2014).

115. Kierdorf, K. et al. Microglia emerge from erythromyeloid precursors via Pu.1- and Irf8-dependent pathways. *Nat. Neurosci.* **16**, 273–280 (2013).

116. Nord, A. S., Pattabiraman, K., Visel, A. & Rubenstein, J. L. R. Genomic perspectives of transcriptional regulation in forebrain development. *Neuron* **85**, 27–47 (2015).

117. Yuan, F. et al. Efficient generation of region-specific forebrain neurons from human pluripotent stem cells under highly defined condition. *Sci. Rep.* **5**, 18550 (2015).

118. Kundaje, A. et al. Integrative analysis of 111 reference human epigenomes. *Nature* **518**, 317–330 (2015).

119. Lee, D. S. et al. Simultaneous profiling of 3D genome structure and DNA methylation in single human cells. *Nat. Methods* **16**, 999–1006 (2019).

120. Chander, P., Kennedy, M. J., Winckler, B. & Weick, J. P. Neuron-specific gene 2 (NSG2) encodes an AMPA receptor interacting protein that modulates excitatory neurotransmission. *eNeuro* **6**, ENEURO.0292-18.2018 (2019).

121. Choksi, S. P., Lauter, G., Swoboda, P. & Roy, S. Switching on cilia: transcriptional networks regulating ciliogenesis. *Development* **141**, 1427–1441 (2014).

122. Nakajima, K. et al. Molecular motor KIF5A is essential for GABA(A) receptor transport, and KIF5A deletion causes epilepsy. *Neuron* **76**, 945–961 (2012).

123. Assali, A., Harrington, A. J. & Cowan, C. W. Emerging roles for MEF2 in brain development and mental disorders. *Curr. Opin. Neurobiol.* **59**, 49–58 (2019).

124. Shi, Y. et al. Functional comparison of the effects of TARPs and cornichons on AMPA receptor trafficking and gating. *Proc. Natl Acad. Sci. USA* **107**, 16315–16319 (2010).

125. Lopez de Armentia, M. et al. cAMP response element-binding protein-mediated gene expression increases the intrinsic excitability of CA1 pyramidal neurons. *J. Neurosci.* **27**, 13909–13918 (2007).

126. Zhou, Y. et al. CREB regulates excitability and the allocation of memory to subsets of neurons in the amygdala. *Nat. Neurosci.* **12**, 1438–1443 (2009).

127. Mattson, M. P. & Camandola, S. NF-κB in neuronal plasticity and neurodegenerative disorders. *J. Clin. Invest.* **107**, 247–254 (2001).

128. Dziennis, S. & Alkayed, N. J. Role of signal transducer and activator of transcription 3 in neuronal survival and regeneration. *Rev. Neurosci.* **19**, 341–361 (2008).

129. Fontenot, M. R. et al. Novel transcriptional networks regulated by CLOCK in human neurons. *Genes Dev.* **31**, 2121–2135 (2017).

130. Pickrell, J. K. Joint analysis of functional genomic data and genome-wide association studies of 18 human traits. *Am. J. Hum. Genet.* **94**, 559–573 (2014).

131. Maurano, M. T. et al. Systematic localization of common disease-associated variation in regulatory DNA. *Science* **337**, 1190–1195 (2012).

132. Wakefield, J. Bayes factors for genome-wide association studies: comparison with P-values. *Genet. Epidemiol.* **33**, 79–86 (2009).

133. Ishii, K., Kubo, K. I. & Nakajima, K. Reelin and neuropsychiatric disorders. *Front. Cell. Neurosci.* **10**, 229 (2016).

134. Carvalho-Silva, D. et al. Open Targets Platform: new developments and updates two years on. *Nucleic Acids Res.* **47** (D1), D1056–D1065 (2019).
