# Supplementary material for: An atlas of gene regulatory elements in adult mouse cerebrum
Source: Nature. 2021 Oct 6;598(7879):129–36. doi: 10.1038/s41586-021-03604-1 (PMC8494637; doi:10.1038/s41586-021-03604-1)
Supplement: Supplementary file 2 — Reporting Summary [file 41586_2021_3604_MOESM2_ESM.pdf]

nature research

Corresponding author(s): Bing Ren

Last updated by author(s): Apr 17, 2021

Reporting Summary

Nature Research wishes to improve the reproducibility of the work that we publish. This form provides structure for consistency and transparency in reporting. For further information on Nature Research policies, see Authors & Referees and the Editorial Policy Checklist.

Statistics

For all statistical analyses, confirm that the following items are present in the figure legend, table legend, main text, or Methods section.

n/a	Confirmed	
☐	☒	The exact sample size (*n*) for each experimental group/condition, given as a discrete number and unit of measurement
☐	☒	A statement on whether measurements were taken from distinct samples or whether the same sample was measured repeatedly
☐	☒	The statistical test(s) used AND whether they are one- or two-sided<br>*Only common tests should be described solely by name; describe more complex techniques in the Methods section.*
☒	☐	A description of all covariates tested
☐	☒	A description of any assumptions or corrections, such as tests of normality and adjustment for multiple comparisons
☐	☒	A full description of the statistical parameters including central tendency (e.g. means) or other basic estimates (e.g. regression coefficient) AND variation (e.g. standard deviation) or associated estimates of uncertainty (e.g. confidence intervals)
☐	☒	For null hypothesis testing, the test statistic (e.g. *F*, *t*, *r*) with confidence intervals, effect sizes, degrees of freedom and *P* value noted<br>*Give P values as exact values whenever suitable.*
☒	☐	For Bayesian analysis, information on the choice of priors and Markov chain Monte Carlo settings
☐	☒	For hierarchical and complex designs, identification of the appropriate level for tests and full reporting of outcomes
☐	☒	Estimates of effect sizes (e.g. Cohen's *d*, Pearson's *r*), indicating how they were calculated

Our web collection on statistics for biologists contains articles on many of the points above.

Software and code

Policy information about availability of computer code

Data collection	Sony Cell Sorter Software v2.1.2-5, Biomek Software 5.1 (library preparation) Illumina HiSeq2500 and HiSeq4000 instrument control software (sequencing)
Data analysis	Seurat (v3.1.2), sklearn (v0.22), bwa mem (v.0.7.17), Cicero (v1.3.4.5), HOMER (v4.11), liftOver (http://hgdownload.soe.ucsc.edu/admin/exe/linux.x86_64/), Enrichr (v2.1), GREAT (4.0.4), BEDTools (v2.25.0), ATACseqQC (1.8.5), pvclust (v2.2.0), MACS2 (v2.1.2), fitdistrplus (v1.0.14), LDSC (v1.0.1)<br>https://github.com/yal054/snATACutils<br>https://github.com/r3fang/SnapATAC

For manuscripts utilizing custom algorithms or software that are central to the research but not yet described in published literature, software must be made available to editors/reviewers. We strongly encourage code deposition in a community repository (e.g. GitHub). See the Nature Research guidelines for submitting code & software for further information.

Data

Policy information about availability of data

All manuscripts must include a data availability statement. This statement should provide the following information, where applicable:

- Accession codes, unique identifiers, or web links for publicly available datasets
- A list of figures that have associated raw data
- A description of any restrictions on data availability

Demultiplexed data can be accessed via the NEMO archive at: https://assets.nemoarchive.org/dat-wywv153
Processed data are available on our web portal and can be explored here: http://catlas.org/mousebrain
Additional data are available in the NCBI Gene Expression Omnibus (GEO) under accession number GEO173650 and upon request.

Field-specific reporting

Please select the one below that is the best fit for your research. If you are not sure, read the appropriate sections before making your selection.

☒ Life sciences ☐ Behavioural & social sciences ☐ Ecological, evolutionary & environmental sciences

For a reference copy of the document with all sections, see nature.com/documents/nr-reporting-summary-flat.pdf

Life sciences study design

All studies must disclose on these points even when the disclosure is negative.

Sample size	No statistical methods were used to predetermine sample sizes.
Data exclusions	No samples were excluded.<br>For analysis only nuclei with > 1,000 reads/nucleus and transcriptional start site enrichment > 10 were selected.<br>Potential barcode collisions were excluded from analysis
Replication	Experiments were performed for 2 biological replicates for each dissected region
Randomization	There was no randomization of the samples
Blinding	Investigators were not blinded to the specimens being investigated.

Reporting for specific materials, systems and methods

We require information from authors about some types of materials, experimental systems and methods used in many studies. Here, indicate whether each material, system or method listed is relevant to your study. If you are not sure if a list item applies to your research, read the appropriate section before selecting a response.

Materials & experimental systems

n/a	Involved in the study	
☒	☐	Antibodies
☒	☐	Eukaryotic cell lines
☒	☐	Palaeontology
☐	☒	Animals and other organisms
☒	☐	Human research participants
☒	☐	Clinical data

Methods

n/a	Involved in the study	
☒	☐	ChIP-seq
☐	☒	Flow cytometry
☒	☐	MRI-based neuroimaging

Animals and other organisms

Policy information about studies involving animals; ARRIVE guidelines recommended for reporting animal research

Laboratory animals	Adult C57BL/6J male mice were purchased from Jackson Laboratories.
Wild animals	No wild animals were used in this study
Field-collected samples	No filed-collected samples were used in this study
Ethics oversight	All experimental procedures using live animals were approved by the SALK Institute Animal Care and Use Committee under protocol number 18-00006.

Note that full information on the approval of the study protocol must also be provided in the manuscript.

Flow Cytometry

Plots

Confirm that:

☒ The axis labels state the marker and fluorochrome used (e.g. CD4-FITC).

☒ The axis scales are clearly visible. Include numbers along axes only for bottom left plot of group (a 'group' is an analysis of identical markers).

☒ All plots are contour plots with outliers or pseudocolor plots.

☒ A numerical value for number of cells or percentage (with statistics) is provided.

Methodology

Sample preparation	Nuclei were stained with DRAQ7 (#7406, Cell Signaling)
Instrument	Sony SH800
Software	SH800S software
Cell population abundance	NA
Gating strategy	Potential nuclei were first identified using FSC-Area and BSC-Area. Next doublets were removed based on BSC and FSC signal width. DRAQQ7 postive nuclei with 2n count were sorted

☒ Tick this box to confirm that a figure exemplifying the gating strategy is provided in the Supplementary Information.
